# Designing of a new multi-epitope vaccine against *Leishmania major* using Leish-F1 epitopes: An *In-silico* study

**DOI:** 10.1371/journal.pone.0295495

**Published:** 2024-01-02

**Authors:** Mahsa Rabienia, Nahid Mortazavidehkordi, Zahra Roudbari, Rasoul Daneshi, Abbas Abdollahi, Mohammad Yousefian Langeroudi, Esmaeil Behmard, Akbar Farjadfar

**Affiliations:** 1 Department of Medical Biotechnology, Fasa University of Medical Sciences, Fasa, Iran; 2 Department of Medical Parasitology, Fasa University of Medical Sciences, Fasa, Iran; 3 Department of Animal Science, Faculty of Agriculture, University of Jiroft, Jiroft, Iran; 4 Department of Medical Microbiology, Fasa University of Medical Sciences, Fasa, Iran; 5 Noncommunicable Diseases Research Center, Fasa University of Medical Sciences, Fasa, Iran; 6 School of Advanced Technologies in Medicine, Fasa University of Medical Sciences, Fasa, Iran; The University of Burdwan, INDIA

## Abstract

Cutaneous leishmaniasis (CL) is the most common form of the disease which can cause malignant lesions on the skin. Vaccination for the prevention and treatment of leishmaniasis can be the most effective way to combat this disease. In this study, we designed a novel multi-epitope vaccine against *Leishmania major* (*L*. *major*) using immunoinformatics tools to assess its efficacy *in silico*. Sequences of Leish-F1 protein (TSA, Leif, and LMSTI1) of *L*. *major* were taken from GenBank. The helper T (Th) and cytotoxic T (Tc) epitopes of the protein were predicted. The final multi-epitope consisted of 18 CTL epitopes joined by AAY linker. There were also nine HTL epitopes in the structure of the vaccine construct, joined by GPGPG linker. The profilin adjuvant (the toll-like receptor 11 agonist) was also added into the construct by AAY Linker. There were 613 residues in the structure of the vaccine construct. The multi-epitope vaccine candidate was stable and non-allergic. The data obtained from the binding of final multi-epitope vaccine-TLR11 residues (band lengths and weighted scores) unveiled the ligand and the receptor high score of binding affinity. Moreover, *in silico* assessment of the vaccine construct cloning achieved its suitable expression in *E*. *coli* host. Based on these results, the current multi-epitope vaccine prevents *L*. *major* infection *in silico*, while further confirmatory assessments are required.

## 1. Introduction

The worldwide outbreak of leishmaniasis, which has endangered 98 countries and a population of about 350 million people over the years, has led to many efforts to solve this health crisis [1]. Unfortunately, despite many studies about leishmaniasis, there is still no definitive treatment for this zoonotic disease in human [2, 3]. Drugs such as pentavalent antimony (Sb^5+^), pentamidine, amphotericin B and miltefosine are used to combat against types of the disease, but due to high cost and side effects, they cannot be prescribed with confidence [4–6]. In addition, many vaccines were successful *in vitro*, but their effectiveness has not been confirmed in later stages of research and few vaccines such as LEISH-F1 and ChAd63KH DNA vaccine have entered into clinical trials [7, 8].

Understanding the host defense mechanism against the parasite is essential in the development of an effective vaccine. Studies on leishmaniasis have demonstrated that provocation of cellular immunity is essential for defense against the parasite [9]. Once the parasite initiates the host infection, macrophages M1 or M2 play a key role in the parasite’s defense or survival. Following the T helper 1 (Th1) response elicit, the cytokines IL-12, IL-2, IL-17, IFNγ and TNF-α are increased. The presence of these cytokines, especially IFNγ, leads to the differentiation of M1 macrophages, the production of nitric oxide (NO) by inducible nitric oxide synthase (iNOS) causing the elimination of the parasite. On the other hand, by directing the immune response to T helper 2 (Th2), cytokines such as IL-4, IL-13, and IL-10 are produced, which inhibit IFN-γ production and differentiate M2 macrophages. This mechanism causes the infection persistence in the body due to the inefficiency of the immune system in parasitic defense [10, 11]. Also, in some studies, an increase in humoral immune response has been demonstrated by IgG2a antibody production to defense against leishmaniasis [12]. Therefore, vaccination aimed at stimulating Th1, Tc, and humoral immune responses can be highly effective in defense against the parasite.

Several *Leishmania* antigens have been used to synthesize a variety of vaccines against the disease, some of which have inferred sufficient immunogenicity *in vitro*. One of these antigens is Leish-F1 containing *Leishmania major (L*. *major)* Thiol-specific Antioxidant (TSA), *L*. *major* stress inducible 1 (LMSTI1), and *L*. *braziliensis* elongation initiation factor (Leif) [13]. The immunogenicity evaluation has been performed using a vaccine containing Leish-F1 antigen with MPL-SE adjuvant and all of them have been successful in clinical trials [14].

These immunogens can be used to make a variety of vaccines, including protein, DNA and mRNA vaccines. As mentioned, the Leish-F1 protein has demonstrated effectiveness with potential of the immune system provocation, making it a prominent candidate for synthesizing other types of vaccines. On the other hand, due to the large size of the genome of this polyprotein, adoption of potential epitopes is reliable. Considering as a candidate for the synthesis of the DNA vaccine against *Leishmania* spp, it can be cloned into the AAV (adeno-associated virus) vectors [15].

Nowadays, with the advancement of immunoinformatics approach, vaccines can be designed *in silico* prior to *in vitro* and *in vivo* development for the efficacy assessment. According to the prediction methods of immunoinformatics, the most suitable epitopes of each immunogens are selected for vaccine design. Thus, it can decrease the cost and duration of vaccine development along with increase of safety, specify, and effectiveness of vaccines [16, 17]. Also, using a multi-epitope instead of a whole protein vaccine reduce allergic reactions enhancing a protein vaccine efficacy [18, 19]. Moreover, due to the lack of living infectious agents in the design these multi-epitope vaccines [20, 21], the risk of infection among vulnerable individuals is substantially mitigated in this preventive approach [22].

In this study, for the first time, we designed a multi-epitope vaccine using Leish-F1 immunogenic antigen of *L*. *major* to combat against the CL using immunoinformatics tools. To increase the immunogenicity of the vaccine, we also used the profilin adjuvant, the toll-like receptor 11 (TLR11)-agonist, which can interact with the macrophages and monocytes enhancing their recruitment and activation. For this purpose, the most suitable HTL and CTL epitopes of Leish-F1 were predicted and linked by AYY and GPGPG linkers. Also, Profilin with the AAY linker was added to the selected epitopes and predicted in terms of three dimensional (3D) structure validity and refinement, and B-cell epitopes prediction. Furthermore, molecular docking of final vaccine and TLR11, molecular dynamics (MD) simulation and codon optimization were performed.

## 2. Materials and methods

### 2.1 Protein sequence of Leish-F1 polyprotein

The amino acid sequences of leish-F1 polyprotein containing TSA, LmSTI1, and Leif proteins with accession numbers (AF044679, MH061281, and W5XL77) and also profilin (PDB ID: AAX33672.1) as an adjuvant were obtained from GenBank, protein database of the National Center for Biotechnology Information (NCBI, http://www.ncbi.nih.gov/genbank/).

### 2.2 T-cell epitope prediction

To predict the CTL and HTL epitopes of TSA, LMSTI1, and Leif proteins, the IEDB server was used. Prediction of 9-mer and 15-mer epitopes to bind to MHCI and MHCII were done by using IEDB (http://tools.iedb.org/mhcii/). The server predicts the CTL and HTL epitopes on the basis of SMM (stabilized matrix method), Artificial Neural Networks (ANN) and, Allele specific affinity cutoff (IC50) [23]. In this study, we selected the mouse alleles to bind the MHCI and MHCII.

### 2.3 IFN-γ inducing epitope prediction

Using IFN epitope server (http://crdd.osdd.net/raghava/ifnepitope/), prediction of gamma-interferon inducing epitopes of HTL epitopes was performed. The server predicts the epitopes based on the methods such as SVM based, motif based, and hybrid approach. On this server, IFN**-γ** inducing and non-inducing MHC-II binders were determined.

### 2.4 Construction of final multi-epitope vaccine

Using the *in silico* information, the final multi-epitope vaccine was designed. First, AYY and GPGPS flexible linkers were used to join HTL and CTL epitopes, respectively [24]. Then, to enhance the immunogenicity of final multi-epitope vaccine, profilin was added as an adjuvant to the N-terminal of the construct using AYY linker.

### 2.5 Conformational and linear B cell epitope prediction

According to the 3-D structure of the construct, the ElliPro suite (http://tools.iedb.org/ellipro/) was used to predict the conformational and linear B cell epitopes of the final vaccine construct [25]. The server assigns a score to each predicted epitope, named PI (Protrusion index). Firstly, the three-dimensional structure of the protein is estimated by a number of spherical ellipses, which the higher the percentage, the fewer residues outside the ellipsoid. The PI value is defined based on the center of mass remaining outside the largest possible ellipsoid and residues with higher scores have more access to the solvent. Discontinuous epitopes are characterized by PI values and clustered by R distance (in Å between remaining mass centers). The higher the R value, the larger the epitopes.

### 2.6 Prediction and validation of protein structures of TLR11 and final multi-epitope vaccine

AlphaFold2 program was used to predict of tertiary structure of the TLR11 and final vaccine construct, based on a deep neural network algorithm [26]. PROCHECK (http://servicesn.mbi.ucla.edu/PROCHECK/), ProSA (http://prosa.services.came.sbg.ac.at/prosa.php), and ERRAT (http://services.mbi.ucla.edu/ERRAT) servers were used to validate the 3D structure of the TLR11 and final vaccine construct [27]. The PROCHECK gave a Ramachandran plot and assessed the stereochemical quality of the refined model [28]. Furthermore, the ProSA provided an overall quality score based on Z-score value. It should be noted that scores outside the range of native proteins are not acceptable [29].

### 2.7 Evaluation of physico-chemical properties of the final vaccine construct

ProtParam (http://web.expasy.org/protparam/), an online web server was used to calculate the physicochemical characteristics of final multi-epitope vaccine containing aliphatic index (AI), instability index, theoretical PI (isoelectric point) molecular weight, *in vitro* and *in vivo* half-life, and grand average of hydropathicity (GRAVY) [30]. Then, the solubility of the final vaccine construct over expression in *E*. *coli* was calculated using SOLpro (http://scratch.proteomics.ics.uci.edu/) [31].

### 2.8 Allergenicity and antigenicity assessment of the final vaccine construct

AllerTOP v2.0 and AllergenFP1.0 were applied to determine the antigenicity of the final vaccine construct. Using five E-descriptors, including amino acid hydrophobicity, size, amino acid tendency to helix, amino acid abundance, and tendency of β-strand formation, the amino acids in the protein sequence are described in the data set of AllergenFP1.0 server. Also, based on auto-cross covariance (ACC) transformation, the strings were transformed into uniform vectors. The server categories the proteins in two allergen and non-allergen, according to Tanimoto coefficient [32]. AllerTOP v2.0 also used the same five aforementioned E-descriptors. This server classifies the protein to allergen and non-allergen based on k-nearest neighbor algorithm (kNN, k = 1) [33]. Thereafter, the determination of antigenicity was done using ANTIGENpro (http://scratch.proteomics.ics.uci.edu/) and VaxiJen v.2 (http://www.ddgpharmfac.net) servers. ANTIGENpro is an alignment-free and sequence-based server. The accuracy of this pathogen independent server is 82% [34]. Protective antigens were also predicted by VaxiJen, a first alignment-independent prediction [35].

### 2.9 Docking analysis of vaccine construct and TLR11

ClusPro server available at (http://cluspro.bu.edu/login.php) was applied to determine the affinity of binding between ligand (final multi-epitope vaccine) and receptor (TLR11). Elimination of unstructured protein regions, use of attraction or repulsion, calculation of pairwise distance restraints, and homo-multimers construction, are some of the parameters that this server considers to provide docking results of two proteins. In addition, the selection of highly populated cluster centers of low-energy structures is another feature of this server [36]. Finally, visualization of docked complex was performed by PyMOL program.

### 2.10 Molecular dynamics simulation details

The structural properties of the TLR11, multi-epitope vaccine, the final vaccine construct-TLR11 and the TLR11-adjuvant (as positive control) complexes were evaluated in the *in silico* conditions through MD simulation. In this step, Using GROMOS 54a7 and 43a1 package, docked complex of final vaccine and TLR-11 was simulated during 50000 picoseconds (Ps) at 310° K and 1 bar pressure. A Cubic box with periodic boundary conditions with water molecules of SPC/E was also used. Moreover, to minimize the system energy and relaxation of solvent molecule, the steepest-descent algorithm was applied. Also, fixation of the atom was performed by using A LINear Constraint Solver (LINCS) algorithm and SETTLE, an analytical algorithm, was used for solvent molecules. To calculate of total electrostatic energy and other non-bonded interactions, Particle Mesh Ewald (PME) summation method and L-J model (with 10 A° cutoff distances), were used, respectively. Moreover, the temperature and pressure of each system, at the time of coupling equal to 0.1 Ps were preserved using Berendsen weak coupling algorithm. Finally, the root mean square deviation (RMSD), root mean square fluctuation (RMSF), hydrogen bond (H-bond), and radius gyration (Rg) diagrams were plotted for both systems.

### 2.11 *In silico* cloning of final multi-epitope vaccine

For this purpose, SnapGene tool was applied and cDNA was provided as an output of the server. The cDNA sequence was used to determine the CAI value and GC content. The CAI value higher than 0.8 and GC content between 30–70% are acceptable. Then, *XhoI* and *NdeI* restriction enzymes were added to the cDNA sequence. Finally, codon optimization into the pET28a (+) vector was done.

## 3. Results

### 3.1 T-cell epitope prediction

Using IEDB server, CTL epitopes of leishF1consisting three proteins was predicted. Six epitopes were predicted from each protein of leishF1. Therefore, a total of 18 epitopes with the lowest percentile rank was selected. Also, a total of nine HTL epitopes of all proteins was selected and for each protein three epitopes were selected (Tables 1 and 2). Moreover, applying IFNepitope server, 12 HTL epitopes were determined to induce of IFN- **γ**. (Table 3).

**Table 1:** Predicted CTL epitope with the lowest percentile rank.

Protein	alleles	Start	End	Sequence	Percentile rank
TSA	H2-Db	48	56	FSFVCPTEV	1.00
	H2-Dd	51	59	VCPTEVIAF	0.30
	H2-Kb	38	46	WVVLFFYPL	0.17
	H2-Kd	81	89	EYAHLQWTL	1.2
	H2-Kk	145	153	NDMPVGRSV	2.00
	H2-Ld	21	29	MPNGSFKKI	1.25
ImST1	H2-Db	395	403	YSNRAAAYI	0.20
	H2-Dd	252	260	KDPNNTLYI	2.00
	H2-Kb	400	408	AAYIKLGAF	0.35
	H2-Kd	258	266	LYILNVSAV	0.4
	H2-Kk	349	357	EEAYIDPEI	0.27
	H2-Ld	253	261	DPNNTLYIL	0.7
Leif	H2-Db	106	114	LALQTAEVI	0.33
	H2-Dd	164	172	RGALRTESL	0.20
	H2-Kb	191	199	QIYEIFRFL	0.42
	H2-Kd	385	393	HYHTQIDEL	1.10
	H2-Kk	239	247	LEGIKQFFI	0.12
	H2-Ld	23	31	RPIPSFDDM	0.16

**Table 2:** Predicted HTL epitopes with the lowest percentile rank.

Protein	alleles	Start	End	Sequence	Percentile
TSA	H2-IAd	95	109	GGLGTMAIPMLADKT	0.23
	H2-IAb	44	58	YPLDFSFVCPTEVIA	4.36
	H2-IEd	82	96	YAHLQWTLQDRKKGG	14.70
ImST1	H2-IAd	310	324	RQRKYEAAIDLYKRA	0.28
	H2-IAb	368	382	YFKEDKFPEAVAAYT	9.46
	H2-IEd	68	82	PNWAKGYVRRGAALH	14.21
Leif	H2-IAd	103	117	TRELALQTAEVISRI	0.28
	H2-IAb	39	53	RGIYSYGFEKPSSIQ	2.18
	H2-IEd	267	281	IAQSVIFANTRRKVD	14.48

**Table 3:** Predicted IFN-γ inducing epitopes.

No	Epitope	Start	End	Score
1	CPTEVAAYVCPTEVI	4	19	0.68
2	AYEYAHLQWTLAAYN	34	49	0.34
3	AYMPNGSFKKIAAYY	58	73	0.05
4	AAYYSNRAAAYIAAY	69	84	0.75
5	AYAAYIKLGAFAAYL	94	109	0.52
6	YIKLGAFAAYLYILN	98	113	0.13
7	PGPGGGLTMAIPMLA	214	229	0.29
8	AHLQWTLQDRKKGGG	258	273	0.65
9	DRKKGGGPGPGRQRK	266	281	0.58
10	PGYFKEDKFPEAVAA	295	310	0.09
11	NWAKGYVRRGAALHG	318	333	0.03
12	EKPSIQGPGPGIAQS	365	380	0.55

### 3.2 Construction of final multi-epitope vaccine

**Fig 1:**
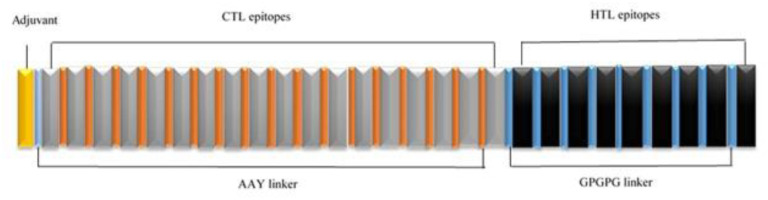
Schematic image of the final multi-epitope vaccine.

A total of 18 CTL epitopes was linked together by AAY linker. Also, GPGPG linker was utilized to join of 9 HTL epitopes with them. Then, profilin with the length of 567 amino acid was added at the N terminal of the construct. The construct was shown in Fig 1.

### 3.3 B cell epitope prediction of final multi-epitope vaccine

**Fig 2:**
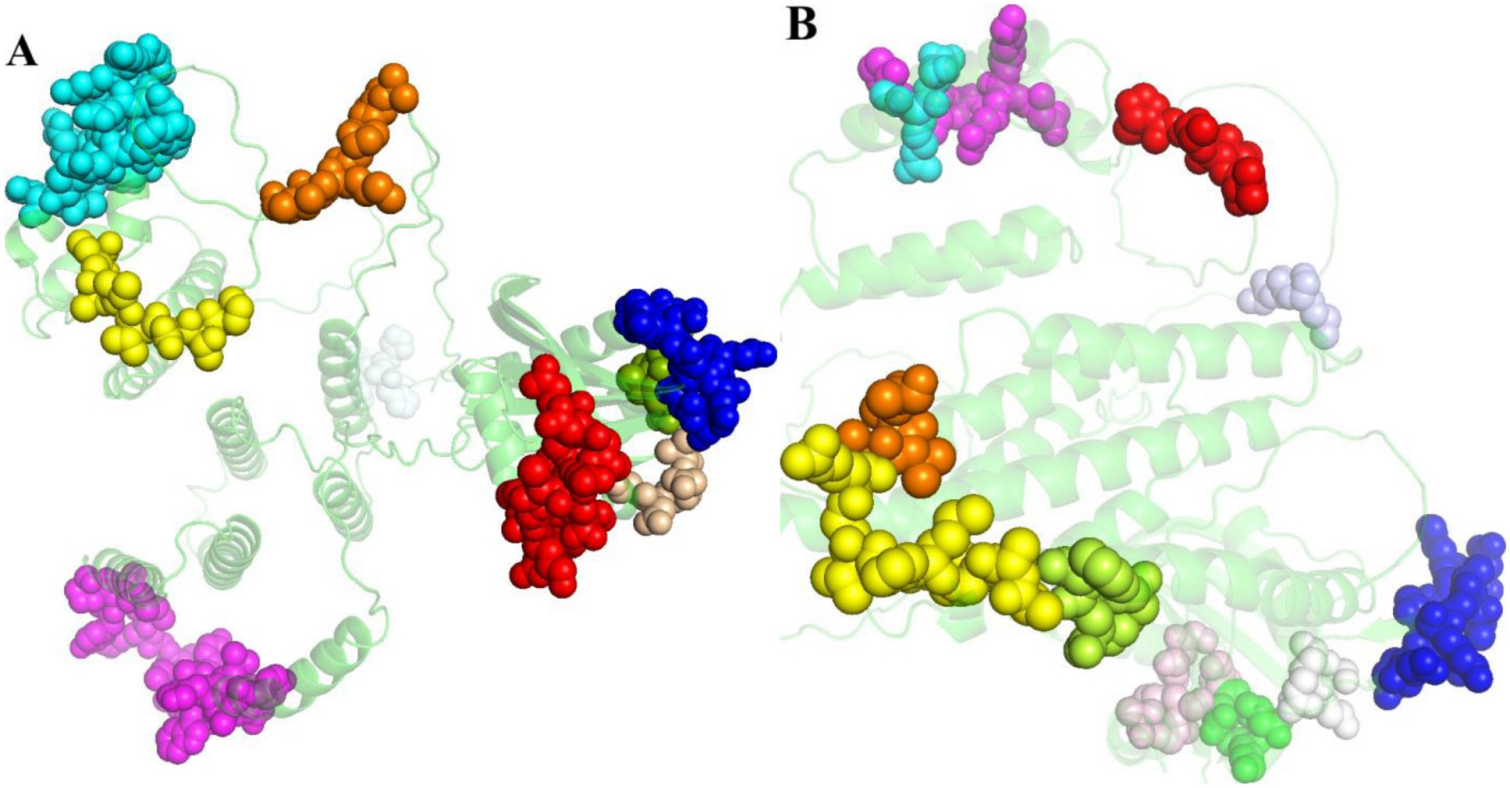
Linear (A) and Discontinuous (B) B-cell epitopes of the construct.

Prediction of linear and discontinuous epitopes of the final vaccine construct was performed using ElliPro suite. According to the results obtained from the server, 9 linear and 11 discontinuous B cell epitopes were predicted in final vaccine with the potential of stimulating humoral immunity (Fig 2A and 2B and Tables 4 and 5).

**Table 4:** Predicted linear B cell epitopes.

No	Start	End	Peptide	Number	Score
1	50	65	HEEDTIGEDGNACGKV	16	0.93
2	206	230	YAHLQWTLAAYNDMPVGRSVAAYMP	25	0.92
3	431	456	QWTLQDRKKGGGPGPGRQRKYEAAID	26	0.90
4	137	146	DEEKEQDKGN	10	0.85
5	416	423	PTEVIAGP	8	0.85
6	37	41	DDDDG	5	0.83
7	14	18	DTGYC	5	0.82
8	564	567	HHHH	4	0.81
9	387	394	GGLGTMAI	8	0.80

**Table 5:** Predicted conformational B cell epitopes.

No	Residues	Number of residues	Score
1	A:A214, A:A215, A:Y216, A:M219, A:P220, A:V221, A:G222, A:R223	8	0.97
2	A:D436, A:R437, A:K438, A:G440, A:G441, A:G442, A:P443, A:G444, A:P445	9	0.95
3	A:E51, A:E52, A:D53, A:T54, A:I55, A:G56, A:E57, A:G59, A:N60, A:A61, A:C62, A:G63, A:K64	13	0.95
4	A:G446, A:R447, A:Q448	3	0.94
5	A:S224, A:V225, A:A226, A:A227	4	0.94
6	A:A207, A:Q210, A:W211	3	0.92
7	A:D137, A:K140, A:Q142	3	0.87
8	A:V419, A:I420, A:A421, A:G422, A:P423	5	0.86
9	A:G390, A:T391, A:M392	3	0.85
10	A:R116, A:S117, A:K118	3	0.85
11	A:T15, A:G16, A:Y17, A:D143, A:G145, A:N146	6	0.82

### 3.4 Prediction and validation of three-dimensional structure of TLR11 and final multi-epitope vaccine

**Fig 3:**
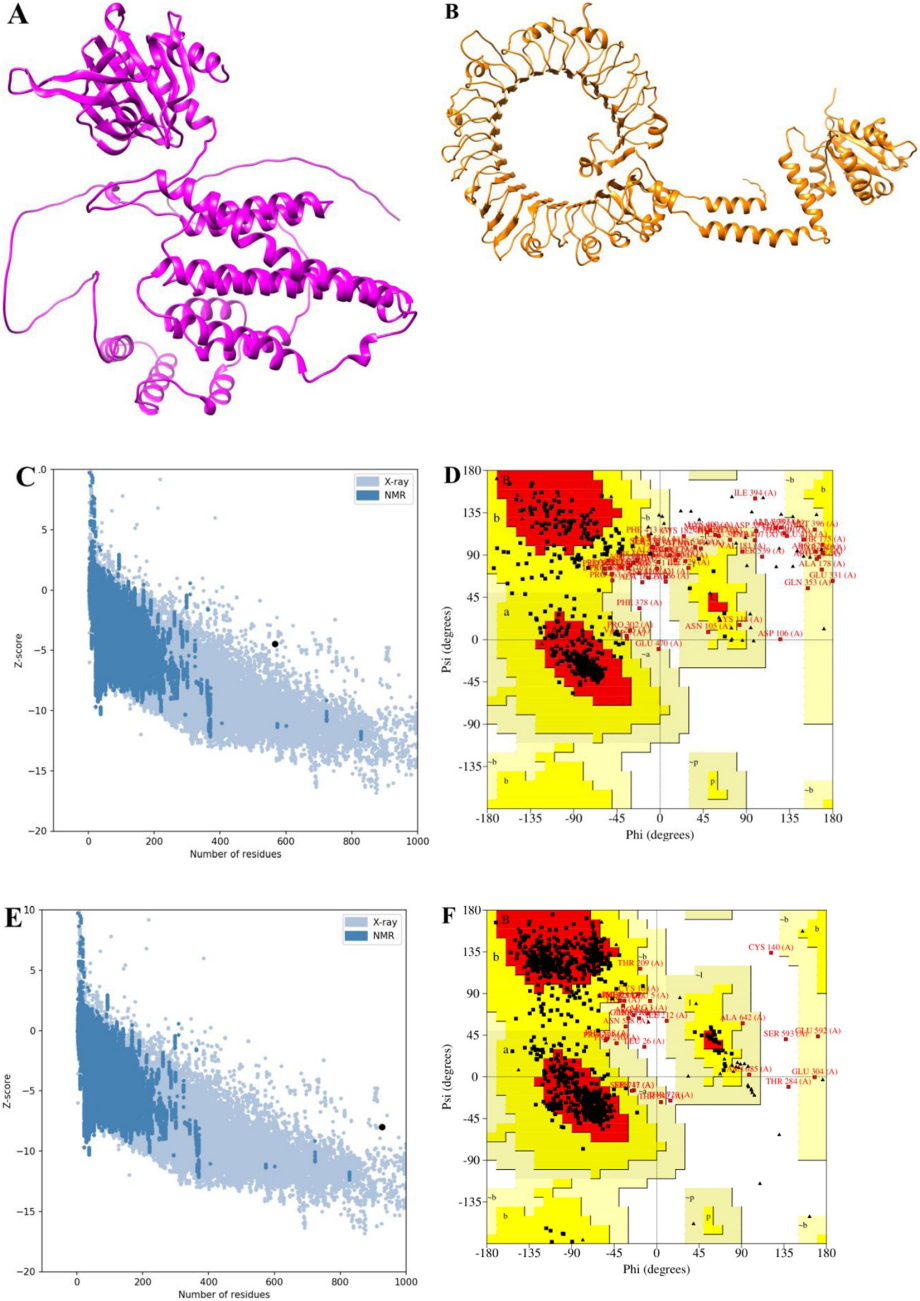
Prediction and validation of the 3D structure of the TLR11 and multi-epitope vaccine; A and B Final predicted models of the vaccine and TLR11 respectively; C and D ProSA and Ramachandran plots for the vaccine predicted model respectively; E and F ProSA and Ramachandran plots for the TLR11 predicted model respectively.

AlphaFold2 results showed five predicted models for the 3D structure of the TLR11 and vaccine. These predicted structures were validated using methods such as the Ramachandran plot, MolProbity, ProSA, and ERRAT. For this purpose, verification of quality and potential errors of the crude 3D models was done. ERRAT method showed values of 81.25% and 80.40% as an overall quality factor of the best models (3A and B), for TLR11 and vaccine respectively (Table 6). The ProSA calculated a Z-score of -4.46, as a plausible value of native proteins as shown in Fig 3C. In addition, the data obtained from the Ramachandran plot of the vaccine exhibited 70.7% residues located in most favored regions, 26.1% residues in allowed regions, and 3.2% in disallowed regions as shown in Fig 3D. The Z-score included -8 for the TLR11 (Fig 3E). Also, in the refined model of the TLR11, 81.1% of the residues were in the favored area, 17.9% in the allowed area and 1.1% in the disallowed area of Ramachandran plot (Fig 3F).

**Table 6:** Validation factors for TLR11 and final multi-epitope vaccine models.

Predicted model	MolProbity	Clash score	Poor rotamers	Rama favored	ERRAT factor	Z-score	Favoured regions	Allowed regions	Disallowed regions
**TLR11**	**2.87**	**27.77**	**3.31**	**90.15**	**82.85**	**-2.6**	**81.1%**	**17.9%**	**1.1%**
**vaccine**	**3.23**	**17.54**	**7.57**	**72.21%**	**75.24**	**-3.74**	**70.7%**	**26.1%**	**3.2%**

### 3.5 Assessment of physico-chemical characteristics and solubility of the final vaccine construct

ProtParam tool was used to determine the physicochemical properties of the final vaccine construct. The values of theoretical PI, and molecular weight were estimated 5.51 and 61.375 KDa, respectively. The amount of PI revealed the final vaccine construct is acidic in nature. The total number of negatively and positively charge was 62 and 48, respectively. Another data in this step such as half-life in mammalian reticulocytes were 30 hours in mammalian reticulocytes, in vitro, >20 hours in yeast and >10 hours in E. coli and in vivo. Also, instability index and AI were computed 29.17 and 75.22, respectively. The stability and thermostability of the construct in nature was confirmed by the values of attained from instability index and AI. The GRAVY score of -0.191 unveiled the construct hydrophilicity in nature. Then, SOLpro server was utilized to investigate the final vaccine construct solubility and antigenicity. The solubility of the vaccine candidate in E. coli and its antigenicity were confirmed giving values of 0.91 and 0.72 respectively.

### 3.6 Evaluation of allergenicity and antigenicity of final multi-epitope vaccine

Allergenicity assessment was done using AllergenFP1.0 and AllerTOP servers. The results revealed the final vaccine construct non-allergenicity. Moreover, its antigenicity was carried out by VaxiJen v.2 and ANTIGENpro servers. Based on the result of VaxiJen at 0.4 threshold in virus model and ANTIGENpro, the probability of the final vaccine was confirmed by the values of 0.43 and 0.72, respectively.

### 3.7 Docking study between final multi-epitope vaccine and TLR-11

**Fig 4:**
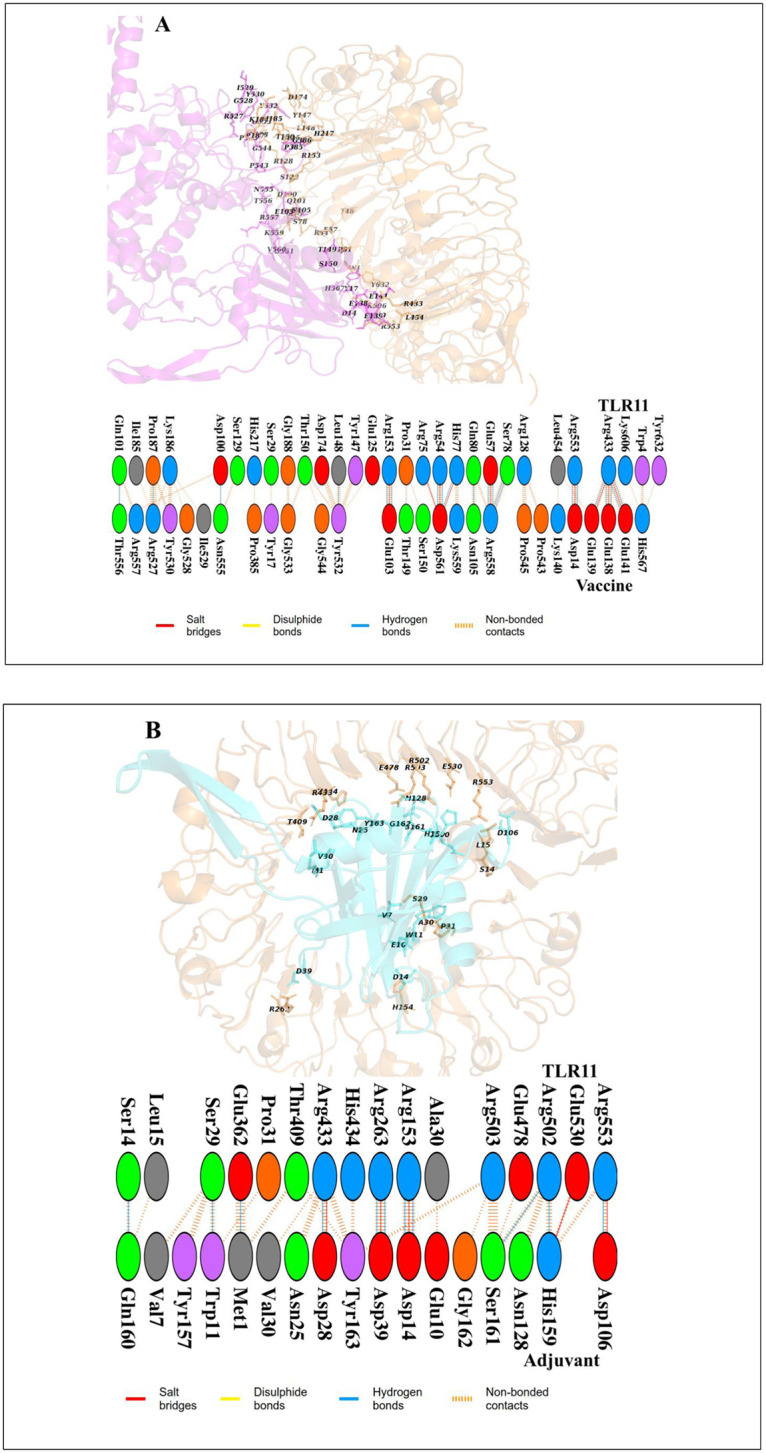
The 3D view and zoom view of the vaccine-TLR11 (A) and adjuvant-TLR11 complexes after MD simulation; The interface residues between each of the vaccine (magenta cartoon) and adjuvant (cyan cartoon) with the TLR11 (brown cartoon) are labeled.

First of all, the MD simulations of the final multi-epitope vaccine and TLR-11 predicted models were performed for 50000 ps, then, ClusPro v. 2.0 webserver was used for the generation of the complex systems. Based on biophysical properties of ligand (multi-epitope vaccine and adjuvant) and receptor (profilin) a total of 30 models were predicted. Among them, the model number 0.00 with the lowest energy score was selected. The weighted score obtained were determined -801.1 (vaccine-TLR11) and -711.5 (adjuvant-TLR11) kcal with the highest binding affinity in all of the predicted models. These complexes were used as initial structures for MD simulation. After 50 ns MD simulation, for a better understanding of the protein-protein interface region of the complex systems, pdbsum web server was used. These analyses show that there are hydrogen bonds, salt bridges, and non-bonded contacts at the interface region of the vaccine-TLR11 and adjuvant-TLR11 complexes (Fig 4A and 4B). Based on this analysis, 9 salt bridges, 21 hydrogen bonds, and 173 non-bonded contacts were found in the interface region of the vaccine-TLR11 complex. Although, 5 salt bridges, 11 hydrogen bonds and 85 non-bonded contacts were found between adjuvant and TLR11 (Fig 4A and 4B).

### 3.8 Molecular dynamics simulation trajectory analysis

**Fig 5:**
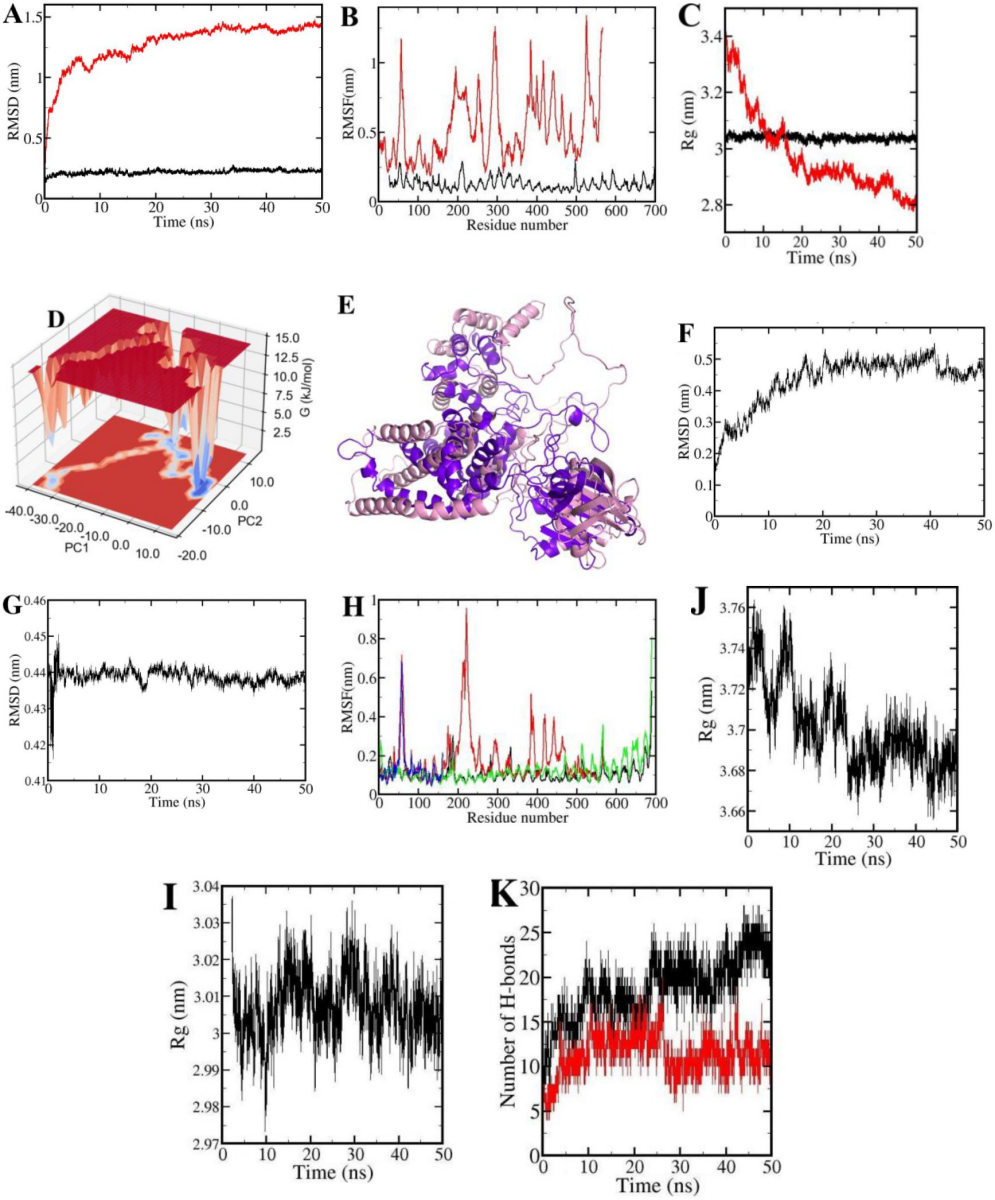
MD simulations results of free (TLR11 and vaccine) and complex (vaccine-TLR11 and adjuvant-TLR11) molecules; (A) the RMSD values of the TLR11 (black line) and vaccine (red line); (B) the RMSF values of the TLR11 (black line) and vaccine (red line); (C) the Rg values of the TLR11 (black line) and vaccine (red line); (D) Gibb’s free energy landscape of the modeled; (E) A representation of the modeled structure (pink cartoon) and spatial configuration (blue cartoon) with the least energy of the vaccine structure. (F) the RMSD values of the vaccine-TLR11 and (G) the adjuvant-TLR11; (H) the RMSF values of the vaccine (red line), adjuvant (blue line) and TLR11 to the vaccine (black line) and adjuvant (green line) complexes; (I and J) the Rg values of the vaccine-TLR11 and adjuvant-TLR11 complexes, respectively; (K) the number of H-bonds between each of the vaccine (black line) and adjuvant (red line) with the TLR11. A-C: free state; F-K: complex state.

The RMSD values for the multi-epitope vaccine and TLR11 in the free state were assessed as a time-dependent parameter for calculating the displacement of α–carbon atoms in the two molecular types during the MD simulations (Fig 5A). In accordance with the RMSD diagrams, the TLR11 has a small fluctuation compared to that of the multi-epitope vaccine. The mean RMSD values for the TLR11 and multi-epitope vaccine were respectively (0.22 ± 0.01) nm and (1.3 ± 0.3) nm. Also, the RMSF values of the TLR11 and the multi-epitope vaccine were estimated separately for analyzing the local structural fluctuations (Fig 5B). High values of the RMSF diagrams refer to loop regions in the protein structures in two proteins. The compactness of the protein structures (TLR11 and vaccine) was estimated using the Rg method. The Rg graph of the vaccine (Fig 5C) presented that over the simulation period from 0 ns to 10 ns with sharp slope decreases from 3.4 nm to 3.0 nm, after that, the graph with a slight slope decreased. This indicated that the multi-epitope vaccine changes its configuration more rapidly in this period. It also can be seen in Fig 5A that the sharp increase of RMSD is in accordance with the curve of Rg when the compactness of the multi-epitope vaccine is low. With the increase of protein compactness, the RMSD graph reaches a plateau. Our results unraveled that the multi-epitope vaccine structure compactness during a simulation time increases which in turn reflects its stability. Rg and RMSD fluctuations of the TLR11 are very tiny which presented the high structural stability. Also, Gibb’s free energy was analyzed to study the conformational stability of the modeled vaccine using the first two eigenvectors (PC1 vs PC2) from the simulation trajectory. According to Fig 5D, the energy conformations of the modeled vaccine were limited to the narrow range of energy basins -40 to 10 kJ/mol on PC1 and -20 to 10 kJ/mol on PC2. The RMSD value between the model protein structure and spatial configuration with the least energy of the vaccine structure was calculated to be 1.06 nm (Fig 5E), which may be attributed to the comparatively lower compactness of the model protein structure in comparison to the spatial configuration exhibiting the minimum energy in the vaccine structure. Alternatively, the presence of loop regions in both structures could also lead to this disparity. Our results inferred that the vaccine structure had low-energy basins (Fig 5E). The low Gibb’s free energy value indicates the good structural stability of the multi epitope vaccine.

The structural stability of the vaccine-TLR11 and adjvant-TLR11 complexes was estimated using the computation of the overall and local fluctuations and Rg of the two systems. The mean RMSD values for the vaccine-TLR11 (Fig 5F), and adjvant-TLR11, (Fig 5G) included (0.43 ± 0.02) nm and (0.43 ± 0.02) nm respectively. Furthermore, the RMSF values of the vaccine, adjuvant and TLR11 at the complex systems were estimated separately for analyzing the local structural fluctuations (Fig 5H). High values of the RMSF diagrams refer to loop regions in the protein structures in complex systems. The high flexibility had no negative effect neither on vaccine and adjuvant binding nor on the total stability of the vaccine-TLR11 and adjvant-TLR11 complex systems.

The value of Rg for the vaccine-TLR11 (Fig 5I) and adjvant-TLR11 (Fig 5J) complexes indicates a decrease along the MD simulation. This means that the compactness of complex systems were increased during MD simulation. Hence, the studied complexes had a proper structural stability.

Our results outlined that compared to the adjvant-TLR11 as positive control, the vaccine complex had stable dynamics with the TLR11 receptor, confirming the strong binding of the vaccine to the TLR11.

### 3.9 Contribution of energy components to the vaccine binding along MD simulations

The interaction energies between each of the vaccine and adjuvant with TLR11 was estimated as separate electrostatic (ΔE_ele_) and van der Waals (ΔE_vdW_) components (Table 7) using MMPBSA approach. According to Table 7, hydrophobic (ΔE_non-polar_) energy term has a favorable role in the binding each of the vaccine and adjuvant to the TLR11. Based on our results, non-polar component plays the major role in the vaccine and adjuvant binding as main driving force (Table 7). Notably, the ΔG_bind_ of the vaccine-TLR11 complex was considerably better than that of adjuvant-TLR11, highlighting stronger and more stable binding and higher affinity of vaccine-TLR11 complex. According to the Fig 5K, the number of H-bonds between the vaccine and TLR11 (Fig 4A and 4B) in the interface regions was higher than those of adjuvant and TLR11 during the simulation time, which also confirmed the complex stability.

**Table 7:** The contribution of various energy components in the ΔG_bind_ (kcal/mol) between vaccine candidate and the TLR11.

Interaction energy	TLR11-vaccine	TLR11-adjuvant
ΔE_ele_^a^	-649.24 ± 20.01	-300.27 ± 13.97
ΔE_vdW_^b^	-145.47 ± 8.36	-98.43 ± 9.10
ΔG_PB_^c^	687.4 ± 31.20	360.65 ± 15.03
ΔG_SA_^d^	-21.27 ± 1.38	-13.78 ± 0.66
ΔE_non-polar_^e^	-166.74 ± 4.87	-112.21 ± 4.83
ΔE_polar_^f^	38.16 ± 26.50	60.38 ± 14.50
ΔG_bind_	-128.57 ± 15.68	-51.83 ± 9.66

^a^Electrostatic connection,

^b^van der Waals connection,

^c^Polar contribution of the solvation effect,

^d^Non-polar contribution of solvation effect,

^e^ ΔE_non-polar_ = ΔE_vdW +_ ΔG_SA_,

^f^ ΔE_polar_ = ΔE_ele +_ ΔG_GB_

### 3.10 *In silico* codon optimization of final multi-epitope vaccine

**Fig 6:**
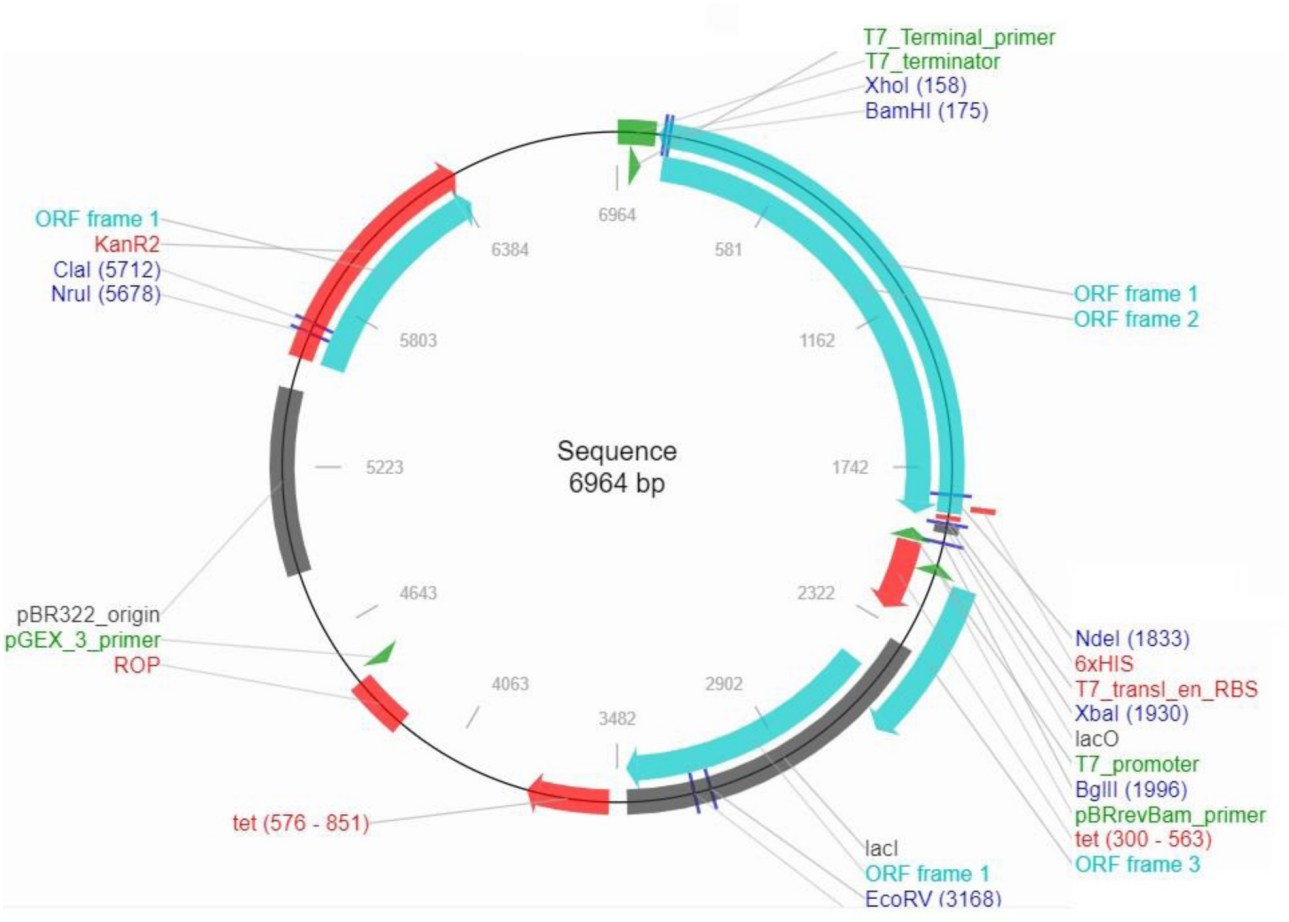
*In silico* cloning of final multi-epitope vaccine construct into pET 28a vector with the length of 6964 bp.

Based on the results of the Java Codon Adaptation tool (JCat), reverse translation and codon optimization in *E*. *coli* (stain K12) was carried out. After the design of cDNA, codon adaptation index and GC percent were calculated 1.0 and 53.35, respectively. The value of GC content was in acceptable rang of the results, being 30–70%. Also the CAI value showed the vaccine highest expression level in the pET28a (+) vector (Fig 6).

## 4. Discussion

There is no definitive treatment for leishmaniasis, in particular the CL form of the disease, which affects 1.5–2 million people annually, and can cause several lesions on the patient’s skin if it progresses, may even lead to the formation of bacterial biofilms and antibiotic resistance in the patient [37–39]. Researchers attempt to make a vaccine for prevention and elimination of the disease [40]. Identifying the host defense pathways against the pathogen and then strengthening these pathways can be the most important strategy in making an effective vaccine. To achieve this goal, immunoinformatics tools can be used to select the most appropriate epitope of the desired immunogens in less time with high accuracy [17, 41].

Leish-F1 is an immunogen and as a protein-based vaccine has already been approved and can be used as a candidate for another type of vaccine, such as a multi-epitope vaccine, which is currently being considered in the prevention of various diseases such as corona virus disease 19 (COVID-19), hepatitis C virus, *Helicobacter pylori*, tuberculosis, Ebola Virus Disease (EVD), Kyasanur forest disease virus (KFDV), coccidiosis, etc [16, 19, 20, 42–47].

The aim of this study was to design a novel multi-epitope vaccine to be effective in combating leishmaniasis. In the first step, HTL and CTL epitopes of Leish-F1 polyprotein from *L*. *major* were predicted and ligated using AYY and GPGPG linkers. Also, due to the fact that in the vaccination against leishmaniasis, the immune system needs to be sufficiently activated, we used the profilin as an adjuvant. The Profilin can bind to TLR11 at the macrophage and dendritic cells surface, leading to innate immune stimulation, increased IFN-γ, and consequently increased Th1. Therefore, profilin can help increase the immunogenicity of this vaccine against *L*. *major*, which was our main goal [48]. There were also 12 epitopes in the final vaccine construct to induce INF-γ, an indispensable cytokine eliciting the immune system response against *Leishmania* spp, these epitopes can be promising for the defense against the parasite.

After the design of the final vaccine construct, conformational and linear B cell epitopes were predicted. The number of twenty linear and conformational epitopes in the structure of the final construct showed that this construct can properly provoke the humoral immunity of the host.

Moreover, tertiary structure prediction of final vaccine construct along with its validation and refinement was implemented. Based on the results of Ramachandran diagram and ProSA, the final vaccine construct was stable in nature. Also the quality and potential errors were predicted using the refinement of final vaccine.

Thereafter, on the basis of physicochemical properties of final vaccine, the PI and GRAVY values included 5.51 and -0.191, respectively, indicating the construct acidity and hydrophilicity in nature. Also, thermostability of the final vaccine was confirmed with the values of 75.22 and 29.17 for AI and instability index, respectively. Then, the solubility of the final multi-epitope vaccine in *E*. *coli* was confirmed.

According to the Rg, RMSD, RMSF and H-bond values obtained from MD trajectory both of the vaccine-TLR11 and adjvant-TLR11 complexes had well structural stability. Additionally, MMPBSA results demonstrated that the hydrophobic residues play a main role in the binding process between each of the vaccine and adjuvant with the TLR11, which in turn maintains the high stability of the complex structures. Therefore, the findings revealed acceptable affinity of the designed multi-epitope vaccine to the TLR11, exhibiting high complex structural stability compared to the adjvant-TLR11 as positive control. The size of the vaccine construct is higher that adjuvant, therefore, more residues interact to the TLR11, and the difference in the interface residues is the result of vaccine and adjuvant differences.

In many vaccine design studies, docking and MD simulations are performed to ensure the efficacy of the construct. Because by confirming the affinity and stability of ligand-receptor binding in conditions similar to the body environment, the efficacy of the vaccine in the *in silico* conditions is confirmed. Finally, the results of codon optimization (CAI value and GC content) showed that the multi-epitope vaccine could have the highest expression in the host.

## 5. Conclusion

In this study, using immunoinformatics tools, a novel multi-epitope vaccine was designed consisting the Leish-F1 of *L*. *major*. The vaccine construct was designed using the most suitable epitopes of cellular immune stimulation of the host and adding an adjuvant (TLR11 agonist) to increase its immunogenicity. The B cell results showed that there are epitopes of humoral immune stimulation in the final construct since humoral immunity can be also effective in defense against leishmaniasis. Moreover, molecular docking and the MD simulation analysis of final multi-epitope vaccine and TLR11 compared to the adjvant-TLR11 as positive control highlighted acceptable binding free energy and confirmed the stability of the vaccine-TLR11 complex. Based on these results, the multi-epitope vaccine was non-toxic and non-allergenic, immunogenic, antigenic, soluble and stable and thereby could stimulate the immune system against *L*. *major*. Despite the vaccine efficiency in the *in silico*, it is necessary to confirm its effectiveness in the further studies *in vitro* and *in vivo*.

## Supporting information

S1 File The predicted three-dimensional structure of TLR11. (PDB)

S2 File The predicted three-dimensional structure of multi-epitope vaccine. (PDB)
